# The lineage-specific, intrinsically disordered N-terminal extension of monothiol glutaredoxin 1 from trypanosomes contains a regulatory region

**DOI:** 10.1038/s41598-018-31817-4

**Published:** 2018-09-12

**Authors:** Mattia Sturlese, Bruno Manta, Andrea Bertarello, Mariana Bonilla, Moreno Lelli, Barbara Zambelli, Karin Grunberg, Stefano Mammi, Marcelo A. Comini, Massimo Bellanda

**Affiliations:** 1 Department of Chemical Sciences, University of Padova, via Marzolo 1, 35131 Padova, Italy; 2 Department of Chemistry “Ugo Schiff”, University of Florence, Via della Lastruccia 3, 50019 Sesto Fiorentino (FI), Italy; 3 Magnetic Resonance Center (CERM), University of Florence, Via L. Sacconi 6, 50019 Sesto Fiorentino (FI), Italy; 4 Centre de RMN à Très Hauts Champs, Institut des Sciences Analytiques (UMR 5280 - CNRS, ENS Lyon, UCB Lyon 1), Université de Lyon, 5 rue de la Doua, 69100 Villeurbanne, France; 5 Department of Pharmacy and Biotechnology, University of Bologna, Viale Giuseppe Fanin 40, 40127 Bologna, Italy; 6 Institut Pasteur de Montevideo, Mataojo 2020, 11400 Montevideo, Uruguay; 7 Laboratorio de Fisicoquímica Biológica, Instituto de Química Biológica, Facultad de Ciencias, Universidad de la República, Igua 4425, 11400 Montevideo, Uruguay; 8 Molecular Modeling Section (MMS), Department of Pharmaceutical and Pharmacological Sciences, University of Padova, via Marzolo 5, Padova, Italy; 9 Present Address: New England Biolabs, 240 County Road, Ipswich, MA 01938 USA

## Abstract

Glutaredoxins (Grx) are small proteins conserved throughout all the kingdoms of life that are engaged in a wide variety of biological processes and share a common thioredoxin-fold. Among them, class II Grx are redox-inactive proteins involved in iron-sulfur (FeS) metabolism. They contain a single thiol group in their active site and use low molecular mass thiols such as glutathione as ligand for binding FeS-clusters. In this study, we investigated molecular aspects of 1CGrx1 from the pathogenic parasite *Trypanosoma brucei brucei*, a mitochondrial class II Grx that fulfills an indispensable role *in vivo*. Mitochondrial 1CGrx1 from trypanosomes differs from orthologues in several features including the presence of a parasite-specific N-terminal extension (NTE) whose role has yet to be elucidated. Previously we have solved the structure of a truncated form of 1CGrx1 containing only the conserved glutaredoxin domain but lacking the NTE. Our aim here is to investigate the effect of the NTE on the conformation of the protein. We therefore solved the NMR structure of the full-length protein, which reveals subtle but significant differences with the structure of the NTE-less form. By means of different experimental approaches, the NTE proved to be intrinsically disordered and not involved in the non-redox dependent protein dimerization, as previously suggested. Interestingly, the portion comprising residues 65–76 of the NTE modulates the conformational dynamics of the glutathione-binding pocket, which may play a role in iron-sulfur cluster assembly and delivery. Furthermore, we disclosed that the class II-strictly conserved loop that precedes the active site is critical for stabilizing the protein structure. So far, this represents the first communication of a Grx containing an intrinsically disordered region that defines a new protein subgroup within class II Grx.

## Introduction

**Figure 1:**
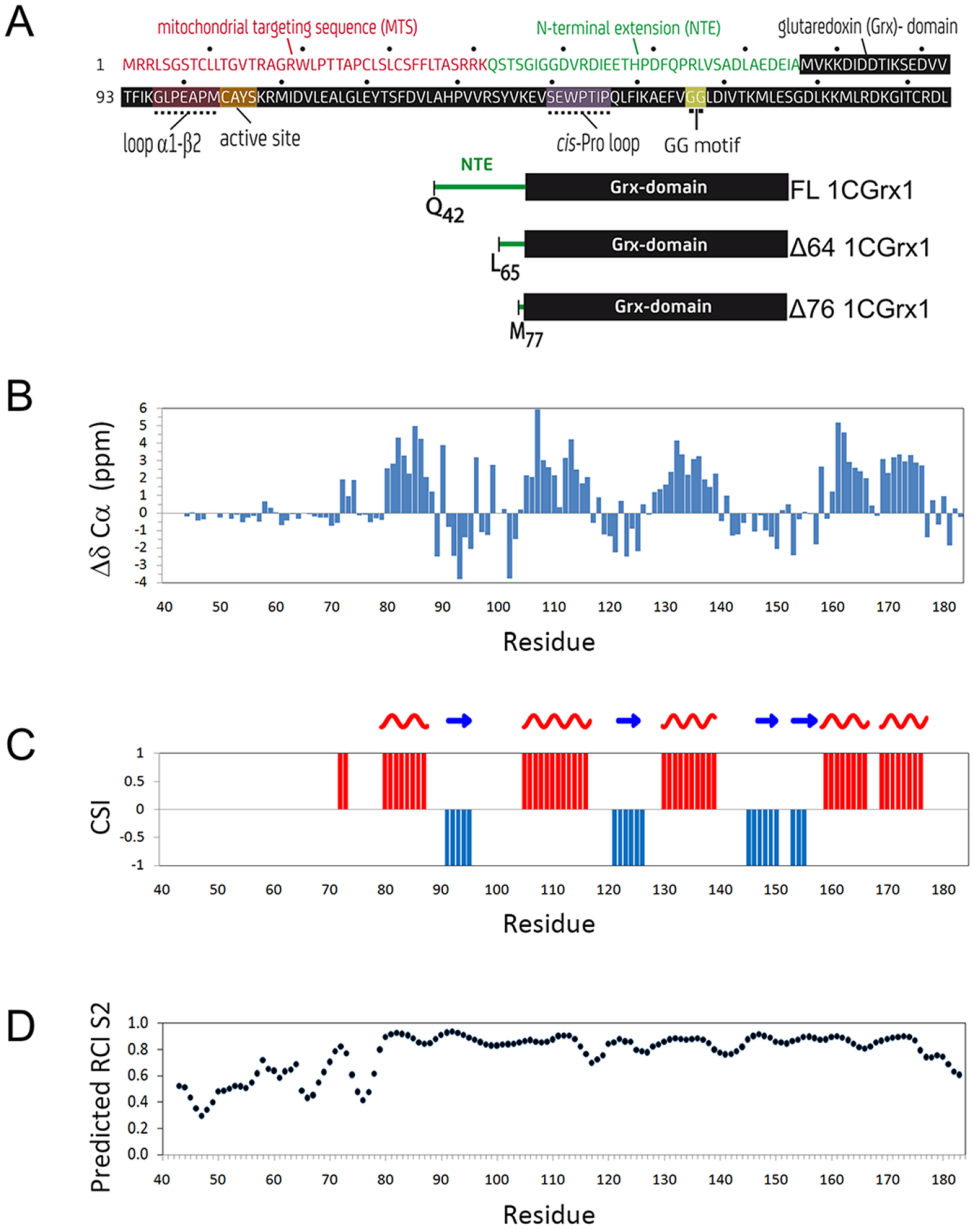
In panel (**A**), the sequence and functional or structural elements/motifs of 1CGrx1 are shown. MTS: mitochondrial targeting sequence, NTE: N-terminal extension and Grx: glutaredoxin-domain. The three forms of 1CGrx1 addressed in this manuscript are schematized and labelled with the corresponding nomenclature: FL 1CGrx1 (residues 42–184); Δ64 1CGrx1 (residues 65–184); Δ76 1CGrx1 (residues 77–184). The additional N-terminal GAMG or GA residues added to FL or Δ76 1CGrx1 respectively, as a consequence of the cloning strategy (see Materials and Methods) are not shown here. In panel (**B–D**), the structural properties of FL 1CGrx1 derived from backbone chemical shift, as a function of residues number are reported (**B**), secondary Cα chemical shift (ΔδCα) (**C**), secondary structure elements predicted using the chemical shift index (CSI) approach: red and blue bars represent α-helix and β-strand respectively (**D**), protein flexibility from the model-free order parameter (S^2^) estimated calculating the Random Coil Index (RCI) from backbone chemical shift.

Glutaredoxins are small proteins involved in the cell redox homeostasis. They are evolutionary conserved and present in most phyla, often with several isoforms and different cellular localizations^1^. This protein family encompasses enzymes with thiol-disulfide oxidoreductase activity, specifically toward protein-glutathione mixed disulfides, and proteins involved in iron homeostasis and iron-sulfur cluster (ISC) biogenesis^2–5^. From a structural point of view, they share a common topological fold, named thioredoxin (Trx) fold, characterized by a four-stranded β-sheet flanked by three to five α-helices^6^. Grx domains are present in more than 100 different protein architectures (148 according to PFAM Grx family PF00462) constituting ~7% of the total diversity of Trx-fold proteins (2187 according to PFAM Trx clan CL0172). The sequence identity among different Grx domains is about 16–37% with less than 15 strictly conserved residues, including a proline in the *cis*-conformation^7^. Despite the relatively low sequence conservation, the overall fold is very similar in all known Grx-domain structures, with backbone RMSD in the range 1.3–2.3 Å^7^. Grx were initially classified as dithiol or monothiol, according to the number of cysteines in the active site. More recently, these two groups were reclassified as class I and class II and four new groups were added^2^. Only class I and II are ubiquitous, while the distribution of the other classes is limited to some kingdoms. Class I Grx generally contain a single domain with one or two cysteines in the active site that display classical oxidoreductase activity. Class II Grx have a highly conserved CGFS motif in the active site (with few exceptions), an insertion of five residues before the active site, a conserved WP motif in a loop containing the *cis*-proline and a GGC motif at the C-term of β4^8^ (Fig. 1A). Class II Grx can exist as single domain proteins or as modular proteins where one or more Grx domains are fused to an N-terminal thioredoxin-like module^9^. With few exceptions, class II Grx are not able to catalyze the reduction of glutathionylated substrates or disulfides. However, most of them can bind labile iron-sulfur (FeS)-clusters and they have been proposed to have an essential role in mediating the transfer of FeS-clusters from scaffold proteins to target apo-proteins^3,5,10,11^. Despite the large amount of publications reported for Grx, the structural homology and the sequence conservation among the members of this family, a defined structure-function relationship has never been established. As a matter of fact, the comparison of the structures available for Grx domains, belonging to either class I or class II, showed a remarkable similarity in the fold and very subtle differences in structure and dynamics of the protein that are likely responsible for the differences in function and selectivity^8,12^.

In its holo-form, namely with the FeS-cluster bound, almost all class II Grx were reported to dimerize using the active site cysteine from each monomer and two molecules of glutathione (GSH) as iron ligands. To our knowledge, the only Grx reported to bind the cluster as a monomer without employing GSH in the coordination is zebrafish Grx2^13^. In most organisms, the GSH/glutathione reductase (GR) pair act concertedly to maintain reduced redox-active Grx at the expenses of NADPH^11^. Some organisms, however, lacks GR and use a different set of proteins and low molecular mass thiols to fuel redox reactions. Among them, protozoan parasites from the order Kinetoplastida are endowed with a thiol-redox system where trypanothione (T(SH)_2_, bis(glutathionyl)spermidine) and the flavoenzyme trypanothione reductase substitute GSH and GR, respectively^14,15^. The uniqueness and essentiality of several components of the redox metabolism of trypanosomatids make them candidates for selective drug design^16,17^.

The genome of trypanosomatids encodes for 2 dithiolic (class I) and 3 monothiolic (class II) Grx^18^. Among them, the class II mitochondrial 1CGrx1 exhibits the most divergent sequence, showing a ~40–60 residues long N-terminal extension (NTE), located between the mitochondrial targeting signal (MTS) and the Grx domain (Fig. 1A), a CA(Y/F)S active site sequence and a C-terminal GG motif that lacks the Cys residue^8,18^. Reverse genetic experiments conducted on *Trypanosoma brucei* (*Tb*) have shown that 1CGrx1 is the only Grx indispensable for the clinically relevant form of the parasite^8,19–21^ and its function is linked to FeS cluster biogenesis^8,22^. The long N-terminal extension conserved among Kinetoplastids has been proposed to promote the non-covalent dimerization of the Grx domain^8,19^. We have previously determined the solution structure of a truncated mutant of *T*. *brucei* 1CGrx1 lacking the first 76 residues (Δ76 1CGrx1; Fig. 1A) and comprising only the conserved Grx domain. In the same paper we showed that Δ76 1CGrx does not bind GSH and T(SH)_2_ and only the protein without the MTS but including the NTE (from here on referred as full-length (FL) 1CGrx1; Fig. 1A) is able to bind the two thiols, although weakly^8^. This observation pointed out that the Δ76 1CGrx1 construct, although designed based on the conservation with homologous Grx domains, is not fully adequate to describe the properties of the protein and that the NTE can influence the binding pocket. More recently we reported the NMR resonance assignment of FL 1CGrx1. The poor dispersion of the peaks in the proton dimension of the HSQC spectrum for residues belonging to this tail, suggested a non-globular nature of the N-terminal extension of the protein^23^. The presence of a long, disordered extension before or after the conserved Grx domain is not a common feature in Grx. Interestingly, a recent analysis of the available genomes of eukaryotes highlighted that the proteome of pathogenic protists, such as *Trypanosoma* and *Leishmania*, is rich in proteins containing disordered regions^24^.

Here, we report on the characterization of *T*. *brucei* FL 1CGrx1 by means of NMR spectroscopy and biophysical approaches. Our study discloses that the NTE is an intrinsically disordered region (IDR). It does not contribute to protein dimerization, as previously proposed, but its portion comprising residues 65–76 has a role in modulating the active site conformation and dynamics. A novel mechanism for the regulation of Grx function, which involves the long-range interaction of an IDR with the conserved GSH-binding residues, is proposed.

## Results

### The N-terminal extension of 1CGrx1 is an intrinsically disordered region

Prior *in silico* analysis of the trypanosomatids’ 1CGrx1 NTE sequences predicted that two prolines in a strictly conserved motif (THP_59_DFQP_63_R - numbering according to *Tb*1CGrx1) split it into two regions that are structurally different. The N-terminal half (G48 to P59) displayed low propensity to adopt a regular secondary structure whereas the C-terminal half (P63 to V75) was predicted to form regular secondary structures^8^. The disordered nature of the N-terminal extension was suggested previously^8,23^ and is conclusively shown here by several new pieces of evidence obtained by NMR. The residues belonging to the NTE show a poor dispersion of the proton chemical shifts in the ^1^H-^15^N HSQC spectrum. Most of these peaks cluster in the region between 8 and 8.5 ppm, with significantly higher intensities than average. The conformational preferences of the NTE can be addressed by a more detailed analysis of backbone chemical shifts. Cα secondary chemical shifts are highly sensitive probes for local conformation^25^; most residues of the tail present absolute values below 0.5 ppm, which is typical of non-structured elements (Fig. 1B). Only residues 72–74 show significant positive secondary shift indicating the formation of a short helical turn separated from the first helix of the globular Grx domain by a short-disordered segment. The secondary structure elements predicted from backbone chemical shifts for the conserved Grx domain (using an improved version of Chemical Shift Index (CSI) method^26^) correspond with those derived from the structure of the 1 CGrx1 mutant lacking the NTE^8^(Fig. 1C). A highly dynamic nature of the tail is indicated also by the Random Coil Index (RCI) analysis^27^, which estimates protein flexibility from chemical shift data. The model-free order parameters estimated with the RCI method show that most residues from the NTE present values consistently lower than the average value of 0.7 of the globular domain (Fig. 1D).

**Figure 2:**
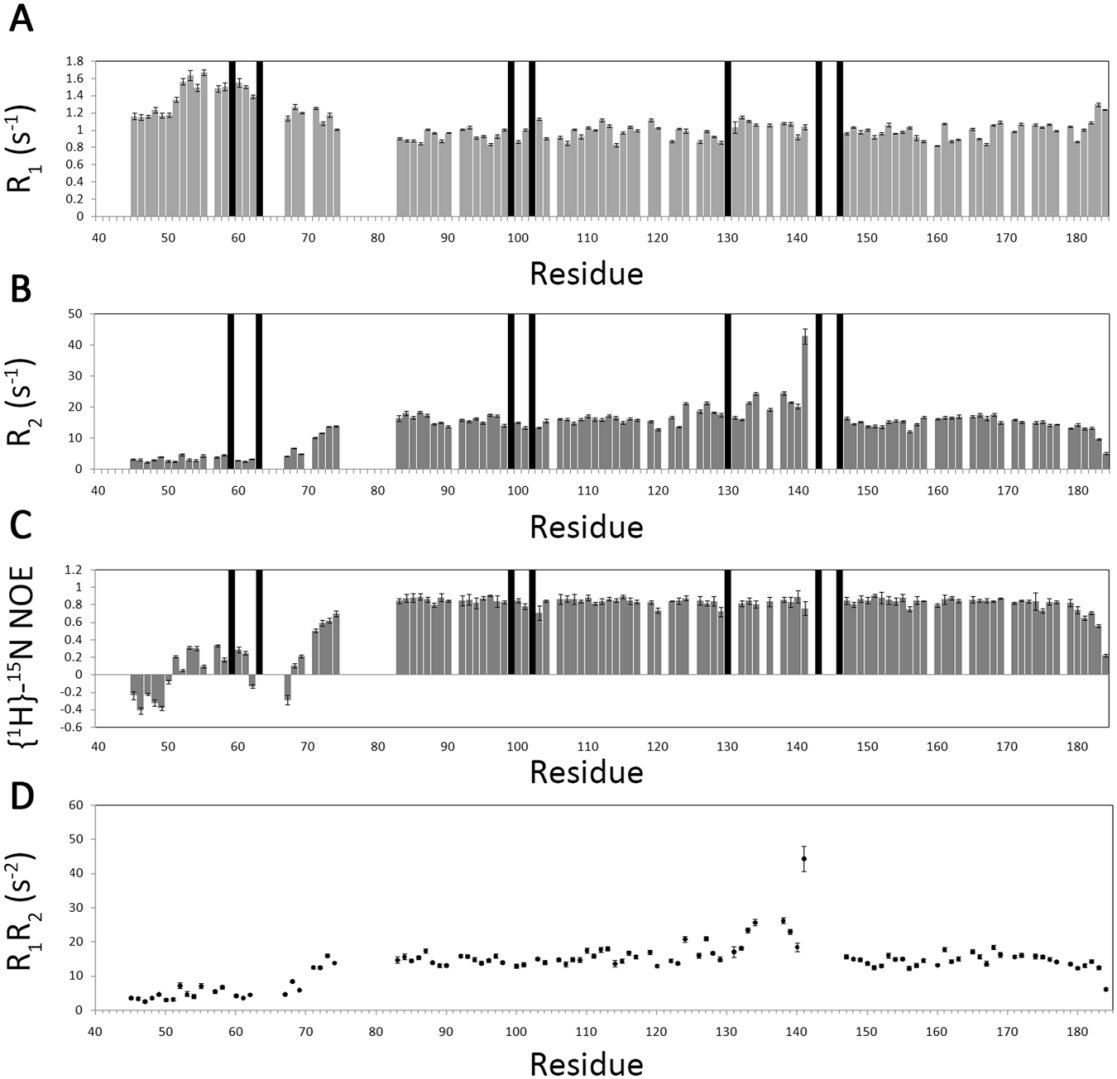
Backbone dynamics of FL 1CGrx1 probed by ^15^N-relaxation rates measured at a proton Larmor frequency of 600 MHz, at 298 K. Residues with severe peak overlap or poor signal-to-noise ration were excluded; proline residues are indicated with black bars. Longitudinal relaxation rate R_1_ (**A**), transverse relaxation rates R_2_ (**B**), steady-state ^1^H-^15^N heteronuclear NOE (**C**) and the product R_1_R_2_ (**D**) are shown as a function of the protein sequence.

An experimental quantification of the dynamic properties of FL 1CGrx1 was obtained from ^15^N relaxation data (Fig. 2). Specifically, heteronuclear-NOE (hetNOE) reports on large-scale fast motion (ns-ps timescale)^28,29^ and low hetNOE values (<0.6) correspond to very flexible portions of the protein (i.e. disordered regions). The average het-NOE value of 0.82 ± 0.09 for the Grx domain confirms that it is very rigid and well folded. In contrast, hetNOE values below 0.4 were obtained for all residues upstream L70 providing additional evidence that the NTE is largely unstructured (Fig. 2C). Interestingly, residues 71–74 showing a propensity to fold in α-helix as described above, present intermediate hetNOE values between 0.5 and 0.7 confirming that this segment of the protein is not fully disordered. Additional evidence supporting this conclusion comes from the residual dipolar couplings (RDC), which contain dynamic information in a timescale different from that described by the relaxation parameters. The plot of ^1^H-^15^N RDCs as a function of the residue number (Fig. S1) confirm that the NTE is largely disordered, presenting for most of the residues RDC absolute values below 3 Hz, hence significantly smaller than the average for the globular domain (7.2 Hz). An important exception is again the segment 71–73 with RDC values above 3.5 Hz.

**Figure 3:**
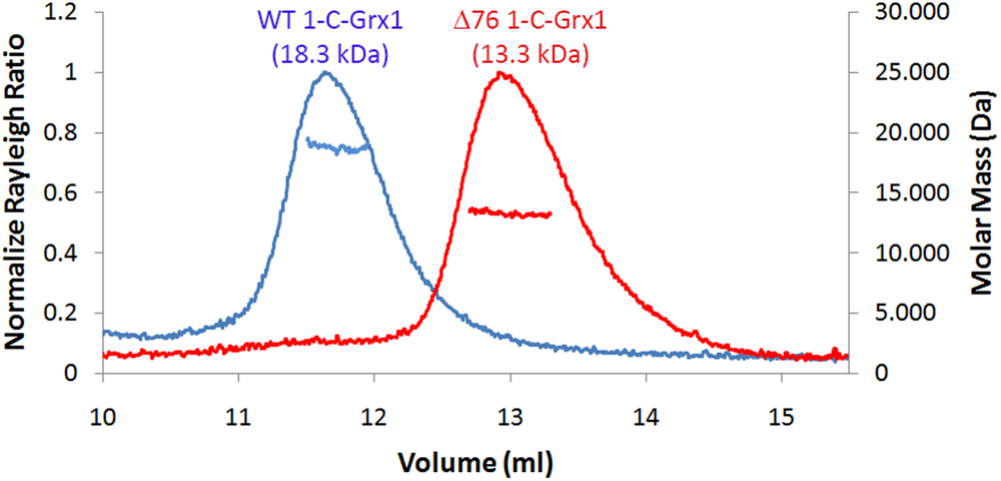
Elution profiles of FL 1CGrx1 (blue) and its Δ76 mutant (red) examined by SEC-MALS. The horizontal blue and red lines correspond to SEC-MALS calculated masses for full-length- and Δ76*-*1CGrx1, respectively. The corresponding theoretical masses are 16.1 kDa (full-length) and 12.3 kDa (Δ76).

Based on size exclusion chromatography (SEC), we have previously proposed that the NTE may promote the formation of non-covalent dimers in apo-1CGrx1^8,19,30^. Considering the IDR nature of the NTE and its potential contribution to an anomalous behaviour of 1CGrx1 in solution, we revisited this hypothesis by applying three complementary experimental approaches. First, as for the Δ76 mutant^8^, ^15^N relaxation measurements were acquired for FL 1CGrx1. The ratio between *T*_1_ and *T*_2_ provides an estimation of the rotational correlation time (*τ*_c_) for the global tumbling of the molecule and this can be related to its molecular mass^31^. After filtering out residues from flexible regions or involved in chemical exchange processes^32^, we estimated *τ*_c_ = 9.5 ± 0.5 ns for FL 1CGrx1, which is fully compatible with a monomeric and not a dimeric state, as previously assumed^30^ (Supplementary Fig. S2). Not surprisingly, this value is significantly larger than the previously measured *τ*_c_ for the Δ76 mutant (7.9 ± 0.6 ns)^8^ due to the expanded size of the protein containing the unstructured NTE. Second, though FL and Δ76 1CGrx1 exhibit very different elution profiles (Fig. 3), SEC coupled to multiangle light scattering (MALS) detection^33^ confirmed that both protein forms are monomeric in solution (see data in Table 1). Finally, we used pulsed field gradient NMR experiments to determine the effective hydrodynamic radius (*R*_h_) of FL 1CGrx1 and verify if it is compatible with that of a species eluting with an apparent molecular mass corresponding to a dimer. *R*_h_ was calculated from the translational diffusion coefficients experimentally determined for FL 1CGrx1: D = (1.37 ± 0.08) × 10^−10^ m^2^/s (see Material and Methods). The *R*_h_ of a protein can be estimated from the number of residues using equations empirically derived for compact (globular) or disordered proteins^34^. According to these empirical equations, the experimental *R*_h_ measured for FL 1CGrx1 could correspond to a fully structured dimeric protein (thus explaining our previous misleading conclusions based on SEC) or, in agreement with all experimental evidence reported here, to a partially disordered monomer. Assuming that the protein is monomeric, from the measured hydrodynamic radius it is possible to calculate for FL 1CGrx1 a compaction factor^34^ of 0.73, which is fully in agreement with the presence of a structured domain of around 100 residues, namely the Grx domain, and a disordered region of about 40 residues, the NTE. The experimental data presented here, together with the physicochemical analysis of the NTE, definitively proves that apo-1CGrx1 is monomeric and that the anomalous behaviour observed in SEC can be ascribed solely to the disordered/extended conformation adopted by the NTE in solution.

**Table 1:** Experimental and calculated molecular mass (M_r_) and hydrodynamic radius (Rh) for FL- and Δ76-1CGrx1.

1CGrx1	M_r_(kDa)		Rh(Å)		
	SEC-MALS	Calculated from sequence	DOSY	Predicted assuming a folded protein^a^	Predicted assuming a disordered protein^b^
FL (N=142)	18.8 ± 0.1	16.1	25 ± 3	20	37
Δ76 (N = 110)	13.3 ± 0.1	12.3	n.a.	18	32

^a^Calculated using the following equation from ref.^34^: ${R}_{h}^{fold}=(4.75\pm 1.11){N}^{0.29\pm 0.02}$.

^b^Calculated using the following equation from ref.^34^: ${R}_{h}^{diso}=(2.21\pm 1.07){N}^{0.57\pm 0.02}$.

### Solution structure of mature 1CGrx1

**Figure 4:**
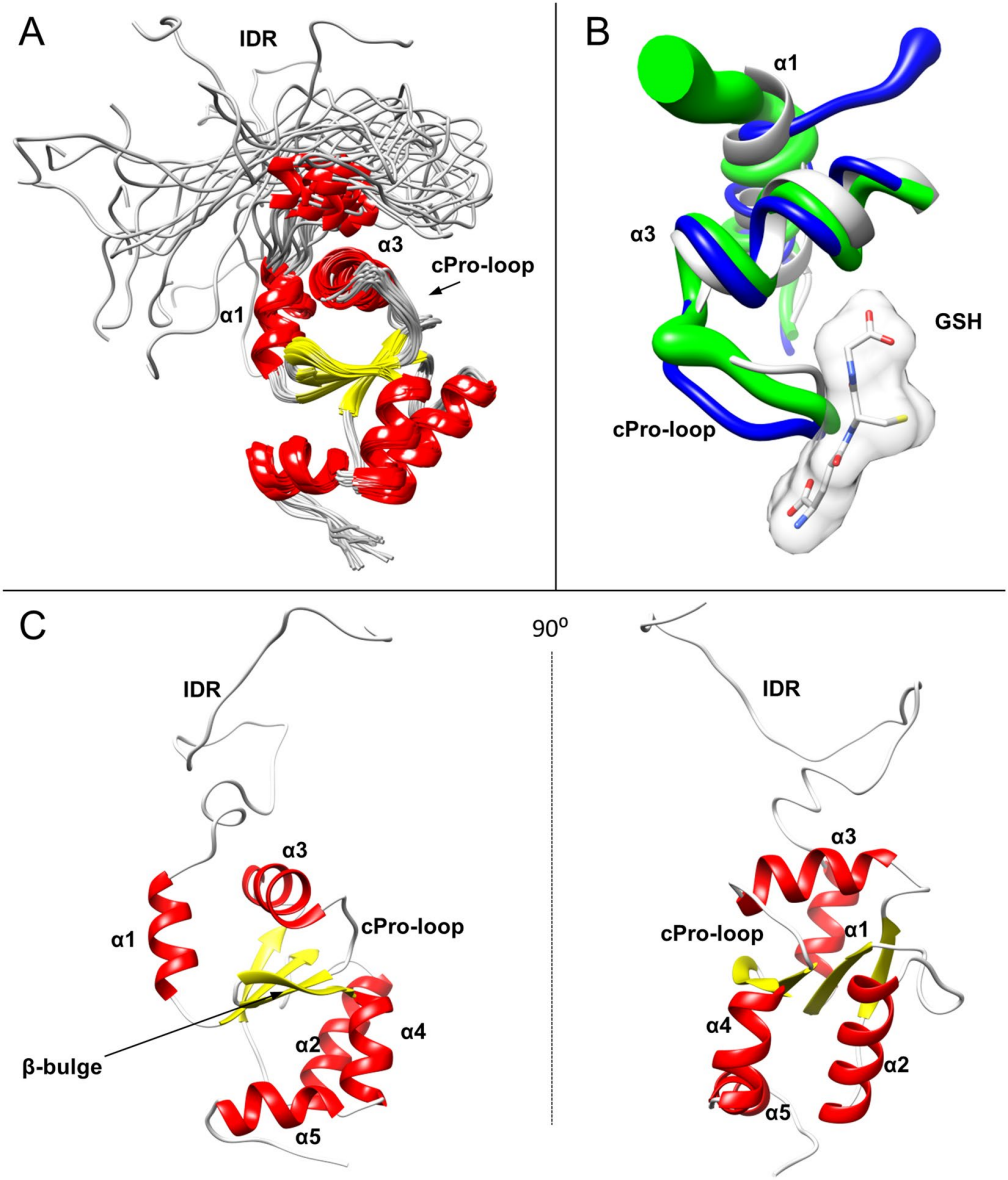
NMR structure of FL 1CGrx1. In panel (**A**), the 20 lowest-energy conformations obtained are reported. The ribbon is coloured in white except for the α-helix and the β-strands, which are in red and yellow, respectively. The reported bundle of conformations is obtained by the superposition of the Grx domain. In panel (**B**), the superposition of FL 1CGrx1 (PDB ID: 2MXN; bundle sausage representation, blue), truncated ∆76 1CGrx1 (PDB 2LTK; bundle sausage representation, green), and the X-ray structure of *E*. *coli* monothiol glutaredoxin Grx4 bound to glutathione (PDB 2WCI; white ribbon and GSH represented both as stick and molecular surface) is reported. The superposition is limited to helix 1, helix 3, and cis-Pro-loop segments. In panel (**C**), the lowest energy structure is reported indicating the position of the α-helix as well as the cis-Pro-loop and IDR.

To gain insight into the interaction of the NTE and the globular Grx domain, the solution structure of FL 1CGrx1 was solved by NMR. The structure was calculated based on NOE derived distance restraints, dihedral angles obtained from TALOS-N^35^ and a set of ^1^H-^15^N residual dipolar couplings^36^ (Table 2). The coordinate file together with the geometrical constraints was deposited in the PDB database (PDB ID: 2MXN). A subset of RDCs not used in the structure refinement was employed to calculate the NMR quality factor^37^ (Table 2 and Supplementary Fig. S3). The superposition of the 20 final lowest energy conformers is shown in Fig. 4A. The N-terminal tail is highly flexible and without a defined secondary structure. The globular, ordered domain spans residues 80 to 183 and displays the typical Trx-fold, characterized by a core of four β-strands surrounded by five α-helices arranged in the order α1-β1-α2-β2-α3-β3-β4-α4-α5. Helices α1 and α3 are located at one side of the β-sheet plane and are almost orthogonal to each other. Helices α2, α4 and α5 are located on the opposite side, where α2 and α4 are essentially parallel. Helices α4 and α5 are almost continuous in the sequence but are structurally orthogonal (Fig. 4C). A classical β-bulge characteristic of the Trx-fold^38^ is present at the end of β4 that, as a consequence, is significantly twisted. Relaxation data clearly show that the folded Grx domain is rigid, with hetNOE values above 0.7 for all residues and an average value of 0.83 (Fig. 2C). The average backbone RMSD for this domain is 1.9 Å but significant differences in the local displacement are evident along the sequence, with residues K80 to D83 and V136 to P143 showing an RMSD larger than the average by at least one standard deviation. Notably, these residues show relatively weak and broad peaks in the HSQC spectrum (not shown), suggesting the presence of chemical exchange associated with conformational plasticity of these regions. Although α1 is locally well folded, its orientation relative to the rest of the domain is not fully defined probably due to the motion of the unstructured NTE. As already discussed above, a helical turn is present downstream to α1 and separated by three residues. Heteronuclear spin-relaxation rates are sensitive, powerful probes of both the overall and the internal dynamics of macromolecules. While the R_2_/R_1_ ratio significantly depends on both motional anisotropy and chemical exchange, it has been demonstrated that the product R_1_R_2_ significantly attenuates the effects of motional anisotropy and represents a sensitive probe of chemical exchange (*R*_*ex*_). As a matter of fact, the R_1_R_2_ values are reduced in the presence of fast motion but, most importantly, they increase in the presence of internal motion in the µs-ms time scale associated with *R*_*ex*_^39^. The plot of R_1_R_2_ as a function of FL 1CGrx1 sequence shown in Fig. 2D clearly confirms the large flexibility of the N-terminal tail and allows the identification of the residues affected by chemical exchange. Residues F124, L127, R133, S134, E138, V139 and E141, all belonging to α3 and the preceding loop, display values at least one standard deviation larger than the average. Remarkably, the R_1_R_2_ values for most other residues from this region and the loop containing the conserved *cis*-proline (D125, Y135, K137, W142, T144, I145), could not be estimated because the peaks were too broad to accurately fit the corresponding R_1_ and R_2_ values from the relaxation experiments. Although the Grx domain of FL 1CGrx1 is almost superimposable to the structure of the NTE-less mutant, the overall precision of both structures is significantly different with an RMSD of 1.9 Å and 0.7 Å, respectively. This difference is in part due to the poorly dispersed intense signals from the NTE that determine ambiguities in the assignment of the NOESY spectrum but mostly to the broadening associated to conformational exchange in α1, α3 and the loop between α3 and β3 (containing the *cis*-proline) (Fig. 4B). Interestingly, these regions embrace residues shaping the GSH binding pocket of class I and II Grx^38^ and, therefore, it is tempting to speculate that the aforementioned conformational dynamics of these regions play a role in regulating the access of ligands to the protein pocket.

**Table 2:** Structure quality report.

NMR Distance & dihedral constraints	
Distance constraints	
Total NOE	1237
Intra-residue (i = j)	276
Sequential (i − j) = 1	454
Backbone-backbone	132
Backbone-side chain	62
Side chain-side chain	260
Medium range 1 < (i − j) < 5	243
Backbone-backbone	82
Backbone-side chain	65
Side chain-side chain	96
Long range (i − j) >= 5	264
Intra-chain	1237
Hydrogen bonds	0
Total dihedral angle restraints	235
Phi	115
Psi	120
Total RDCs	57
Structure Statistics (20 structures)	
Violations (mean.)	
Distance restraints Å	0.07
Dihedral angle restraints (°)	0.76
Max. dihedral angle violation (°)	15.56
Max. distance restraint violation (Å)	0.16
Deviations from idealized geometry	
Bond lengths (Å)	0.024
Bond angles (°)	2.06
Average pairwise RMSD** (Å)	
Heavy (43–184)	7.259
Backbone (43–184)	7.307
Heavy (80–184)	1.755
Backbone (80–184)	1.144
Amber Energy	
Mean constraint energy (kcal/mol)	28.23
Mean AMBER energy (kcal/mol)	−154193.86
RDC	
*Q*	20%

**Pairwise RMSD was calculated among 20 refined structures for the full-length form (NTE + Grx domain; residues 43–184) and for the Grx domain only (residues 80–184).

### The NTE modulates the accessibility to the binding pocket

**Figure 5:**
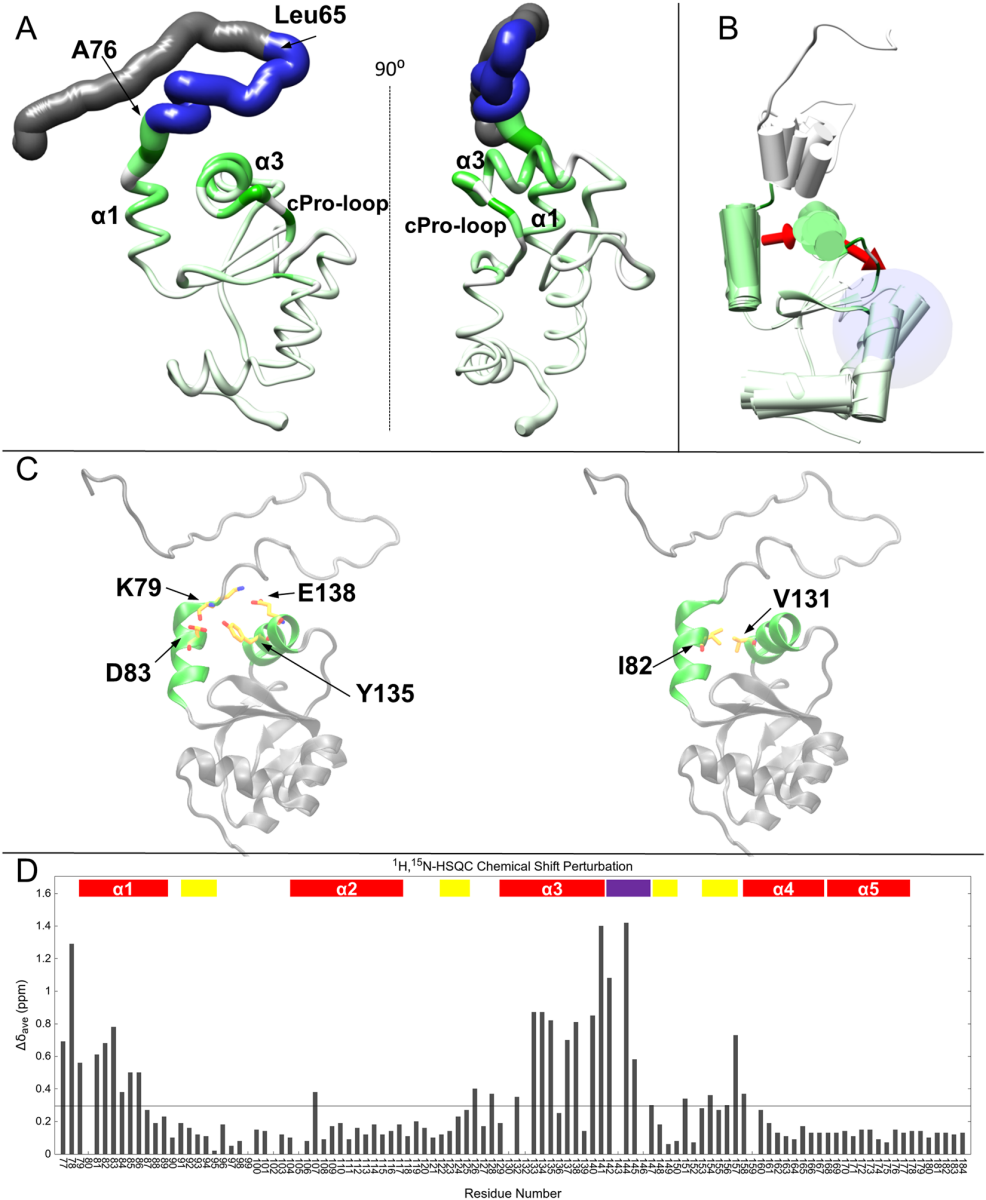
Proposed mechanism for the interaction of the NTE (IDR) with the GSH-binding site. In panel (A), the position of the deletion segment corresponding to Δ64, Δ76 are indicated on the FL 1CGrx1 (AA 42-184) structure. Segment 42–64 is coloured in grey, segment 65–76 is coloured in blue while the globular domain 77–184 is coloured in a green-to-white palette according to the chemical shift perturbations measured comparing the ^1^H-^15^N HSQC of the full-length and truncated Δ76 forms. The green-to-white palette indicates strong-to-none CSPs (see panel D for CSP values). Panel (B) represents a model describing how the fluctuations of the helix α1, due the attached NTE, can be transferred to the GSH-binding site through the interposed helix α3 and can therefore influence its conformation and dynamics. The ribbon is coloured according to the chemical shift perturbations above mentioned. The residues involved in the electrostatic and hydrophobic interactions connecting α1 and α3 (in green) are represented as stick in the 1CGrx1 structures shown in panel (C). In panel (D), the CSPs are plotted in a histogram reporting the averaged perturbation in ppm (∆δ_AVE_) for each residue. The secondary structure elements are reported within the plot while the black line is plotted at a value corresponding to the average CSP value.

Comparison of the ^1^H-^15^N-HSQC spectra of Δ76^8^ and FL 1CGrx1 (this work) revealed shifts in a significant number of peaks, which can be ascribed to structural rearrangements caused by the presence of the NTE. As shown in Fig. 5, the chemical shift perturbation (CSP) is evident not only for α1, to which the NTE is connected, but also for α3 and the downstream loop containing the conserved *cis*-proline. Mapping the CSP on the bundle of conformations for FL 1CGrx1 clearly shows that the regions of the protein most affected by the presence of the NTE correspond to those where the structure is less well defined because of internal mobility, as already described in the previous section (Fig. 5A). Despite the intrinsic flexibility of these regions, it was possible to identify the molecular network responsible to propagate the effect of the conformational flexibility of the NTE to the protein binding pocket (Fig. 5B). Residues K79 and D83 located on α1 are involved in electrostatic interactions with residues Y135 and E138 on α3 while hydrophobic interactions connect residues I82 with V131 and V132 (Fig. 5C). This network of interactions is more precisely defined in the NMR structure of the Δ76 truncated mutant where the atomic positions of α1 and α3 are better defined than in the mature protein^8^. No NOEs were observed between the NTE and the *cis*-Pro loop (W^142^PTIP^146^) excluding a stable, direct interaction with those regions. To understand which part of the NTE is responsible for the CSP on the Grx domain, we performed a limited proteolysis assay of FL 1CGrx1. This technique is also suitable to detect IDR since they are particularly prone to be cleaved by proteases due to their accessibility to the solvent and flexibility^40^. After optimization of the cleavage conditions (for details, see Supplementary Data), trypsin treatment of FL 1CGrx1 yielded protein cleaved between K64 and L65, as confirmed by mass spectrometry (see Supplementary Data), and therefore named Δ64 1CGrx1 (Figs 1B and 5A). Notably, the peak pattern of the ^1^H-^15^N-HSQC spectrum from Δ64 1CGrx1 is essentially identical to that observed for the full-length protein (Fig. S4). This result clearly shows that the segment comprising amino acids L65 to M76, (i.e., the C-terminal part of the NTE), is responsible for modulating the conformational dynamics of FL 1CGrx1. When CSP are compared, it becomes evident that small structural changes at the N-terminus of the Grx domain can be efficiently transferred through α1 and α3 to the putative GSH binding pocket (Figs. 5B and 5D).

Comparison of the crystal structure of the holo-form from the class II Grx from *E*. *coli* (*Ec*Grx4; PDB ID 2WCI)^41^ with Δ76 1CGrx1 led us to propose that a subtle difference in the orientation of the *cis*-Pro loop (W^142^PTIP^146^) may be responsible for the labile binding of low molecular mass thiols by FL 1CGrx1 while Δ76 1CGrx1 does not show any binding ability. Superposition of *Ec*Grx4 (co-crystallized with GSH) with Δ76 and FL 1CGrx1 reveals that in the FL 1CGrx1 structure, the *cis*-Pro loop adopts an intermediate conformation between that of *Ec*Grx4 (open conformation) and apo Δ76 1CGrx1 (closed conformation) (Fig. 4B). In Δ76 1CGrx1, a hydrophobic contact between V136, located at the C-term of α3, and I145 from the *cis*-Pro loop restricts the dynamic of these elements and contributes to occlude the GSH-binding pocket^8^. For FL 1CGrx1, the conformational changes occurring at the C-term of the NTE propagate to α3 with the consequence that the hydrophobic interaction between α3 and the *cis*-Pro loop is lost and the previously reported steric effect of T144 and I145 on the active site of Δ76 1CGrx1 is released. Worth noting, the corresponding *cis*-Pro in *Ec*Grx4 (P72) has been shown to participate in GSH binding via two hydrogen bonds with the γ-Glu moiety of the ligand. Thus, the conformational models of the ligand binding site for FL and Δ76 1CGrx1 are in full agreement with the differences in affinity for low molecular mass thiols previously reported for these proteins^8^.

Altogether, our results support a pivotal role for residues 65-76 of the N-terminal tail of FL 1CGrx1 in modulating the structural dynamic properties of the binding pocket through a network of polar and hydrophobic interactions between residues from α1 and α3.

### The active site loop typical of Class II Grx is indispensable for structure consolidation of 1CGrx1

As pointed out in earlier sections, additional features that distinguish class II from class I Grx are an insertion (consensus motif GxPxxPx) between β1-α2 and a highly conserved WP motif within the *cis*-Pro loop that in the latter class is replaced by a HE motif (see sequence alignment in^19^). It has been speculated that the occurrence of these elements is directly linked to the lack of redox activity in class II Grx^18,42,43^. However, experimental evidence supporting this hypothesis and/or reporting the role of this element in the protein structure and function are missing. To address this issue, two different constructs were generated: in the first one, the active site loop of FL 1CGrx1 (residues G_97_LPEAPM_103_) was replaced by the VT motif of *T*. *brucei* 2CGrx1, a redox-active class I Grx^20^, originating the construct named “loop^(−)^”. In the second one, the conserved WP motif was replaced by HE on the loop^(−)^ 1CGrx1 sequence and the new construct was named “loop^(−)^/HE”. Surprisingly, both constructs formed high molecular weight soluble aggregates, as inferred from the major protein peak eluting at the SEC exclusion volume and from their ^1^H-^15^N-HSQC (Fig. S5), and lacked redox activity (insulin-reduction assay, not shown). These results demonstrate that the active site loop plays an important and more general structural role in the packing of the globular domain of 1CGrx1 than just in modulating the redox activity of class II Grx.

A closer inspection of the relaxation and structural data obtained for FL 1CGrx1 shows conformational restrictions in the loop preceding the active site. These restraints originate in the anchorage of the ends of the Ω-loop (i.e., hydrogen bonds between backbone atoms from K96 and M103) and in the two *trans*-Pro residues of the loop. The decreased end-to-end distance in the active site loop pulls the residues from the active site located on α2 towards the protein core and the *cis*-Pro loop. This structural arrangement differs from that observed for the redox active class I Grx and is likely the basis for the lack of redox activity of class II Grx.

## Discussion

We have previously reported that apo-1CGrx1 exhibits an abnormal elution profile in SEC that nearly correspond to those estimated for a dimeric protein species^8,19,30^. Assuming such oligomeric structure, we concluded that 1CGrx1 is a dimeric protein that changes its conformation but not its oligomeric state upon FeS-cluster binding, clearly differing from other members of class II Grx. Here we demonstrate that the anomalous behaviour of FL 1CGrx1 during gel-filtration can be explained by the presence of an intrinsically disordered region at its N-terminus that increases the hydrodynamic radius of the protein. Although the presence of a long and flexible N-terminal tail is not a common feature in Grx, IDR are particularly abundant in early-branching eukaryotes such as trypanosomatids^24,44,45^. Indeed, 55–70% of the proteins encoded in the genome of trypanosomatids contain IDR larger than 40 amino acids in length, whereas these elements represent only 25% of the human proteome^46^. It has also been suggested that structural disorder may have an important role in mediating protein-protein interactions in pathogenic protozoa with a complex life style^47^. In kinetoplastids, which lack a classical transcriptional regulation of gene expression, disordered elements may play important regulatory roles in protein function to enable the parasites to rapidly adapt to different environmental conditions^24^. The high level of sequence conservation of the NTE in 1CGrx1 from kinetoplastids together with the lack of functional complementation by the NTE-less 1CGrx2^8,19^, strongly suggests that this element is functionally relevant.

The globular domain of FL 1CGrx1 exhibits the typical Trx fold and it is almost superimposable to the structure of known class II Grx^38,41,43,48,49^ and to its Δ76 mutant lacking the disordered NTE. ^15^N relaxation data clearly show that the structure is rigid but several residues from the binding pocket undergo backbone conformational motions as we had already observed for the Δ76 mutant^8^. Banci and coworkers have recently shown that human apo-Grx5 (a canonical class II involved in mitochondrial FeS-cluster biogenesis) exhibits an enhanced structural plasticity in certain regions, which allows the protein to transfer [2Fe-2S] to the target partners^11^. Interestingly, the residues showing higher backbone motion correspond to those highlighted here for FL 1CGrx1. Although the NTE of FL 1CGrx1 does not produce major conformational changes in the protein structure, our data show that its C-terminal portion (residues 65–76) influences the conformation and dynamics of the binding pocket located on the opposite side of the protein. This is the result of a precise network of interactions that connect the NTE-contiguous α1 to the binding site through the interposed α3. It is, therefore, reasonable to assume that structural changes affecting the lineage-specific disordered domain could be transmitted to the binding site affecting its conformational properties and accessibility. As a matter of fact, we have previously shown that while titration of Δ76 1CGrx1 up to very high molar ratios of GSH or T(SH)_2_ did not produce any significant resonance shift in its ^1^H,^15^N-HSQC spectra, identical experiments conducted with FL 1CGrx1 showed small but significant perturbations for several peaks corresponding to residues near the putative GSH-binding pocket^8^. Moreover, our previous experimental evidence indicated that the NTE of FL 1CGrx1, although not critical for ISC binding *in vitro*, contributes to stabilize the holo-complex^8^. Therefore, we can hypothesise that structural changes around residues 65–76 of the NTE, induced by the interaction with a protein partner, may trigger a conformational rearrangement of the binding pocket with functional relevance. Interestingly, our data demonstrate that part of this flexible sequence has indeed a tendency to adopt a helical fold. It is well established that structurally disordered regions could represent a functional advantage for molecular recognition^50^ and there are several examples showing the induced folding of an unstructured portion of a protein upon contact with a folded interacting partner^51^. On this basis, we propose that 1CGrx1 may require a specific partner interacting with the disordered tail, to fulfil its role as part of the mitochondrial FeS-cluster assembly complex. This would explain why, in complementation experiments, the expression of 1CGrx1 in Grx5-deficient yeast cells only partially restored the phenotype of the mutant^30^.

The lack of oxidoreductase activity of class II Grx has been attributed to the presence of an insertion that precedes the active site and exhibits a G_97_xPxxPx_103_ consensus motif (positions according to 1CGrx1 sequence). As shown here for FL 1CGrx1, this element involves conformational restrictions that may propagate to the adjacent residues (i.e., the strictly conserved active site C104 and K96, a charged residue involved in GSH binding) and plays a critical stabilizing role in protein structure since its removal leads to non-covalent protein oligomerization. Interestingly, the features of this loop resemble those of Ω-loops, which are characterized by lack of a defined structure, are relatively rigid and localized on the protein surface where they can be important for protein stability^52^.

In summary, this work provides further understanding of molecular aspects of a class II Grx from a pathogenic organism that harbours a lineage-specific IDR. In this regard, the paradigm that proteins harboring IDR are not suitable drug targets due to conformational heterogeneity and dynamics has been recently challenged by the NMR-based identification of small molecules that bind weakly, but specifically to a disordered protein, and inhibit its activity^53^. Thus, the unique sequence/structural features of 1CGrx1 open the possibility for the discovery of selective inhibitors against this essential protein of trypanosomatids.

## Material and Methods

### DNA constructs

The generation of the plasmids for expression of tag-free 1CGrx1 containing (residues Q42-L184, henceforth “FL”) or lacking the N-terminal extension (residues M77-L184, henceforth “Δ76”) was described in^8,54^. The synthetic open reading frame for 1CGrx1 “loop^(−)^” and “-loop^(−)^/HE” with added KpnI and NcoI restriction sites were obtained from Invitrogen (Life Technologies) and cloned in pGEMT vector (Promega). The fragments for the corresponding ORFs were then obtained by digestion with NcoI and KpnI, run and purified from agarose gel, and used as megaprimers for restriction-free cloning (RF)^55^ using the plasmid for FL 1CGrx1 (pET-trx1b-1CGrx1) as template. Prior to PCR, the parental plasmid was treated with Dam methylase (New England Biolabs) for 20 min at 37 °C. RF PCR reactions (50 μL) were performed using Phusion DNA polymerase, 250 ng of megaprimers and 40 ng of destination vector. PCR cycling was performed as follows: initial denaturation for 30 s at 98 °C, 35 cycles of 10 s at 98 °C, 30 s at 63 °C and 60 s at 72 °C, and a final extension of 3 min at 72 °C. 10 μL of this PCR reaction were digested with 1 μL of DpnI (New England Biolabs) for 1 hour and 30 min at 37 °C to remove parental plasmid. After inactivation of DpnI at 80 °C for 20 min, the reactions were transformed into XL1-Blue competent cells.

### Expression and purification of recombinant proteins

All recombinant proteins were produced as fusion proteins to *E*. *coli* Trx and purified according to the procedure described in^23^. Briefly, transformed *E*. *coli* BL21(DE3) (New England Biolabs) cells were grown at 37 °C, 220 rpm up to a OD_600 nm_ of 0.6–0.8 and protein expression induced overnight at 20 °C, 220 rpm with 0.2 mM IPTG. The unlabeled protein was expressed in LB or 2YT medium, while ^15^N- or ^15^N-^13^C doubly-labelled proteins were produced in M9 minimal medium supplemented with 1 g/L ^15^NH_4_Cl and, 4 g/L glucose or ^13^C-glucose, respectively. Kanamycin (50 µg/mL) was added as selection agent. Cells were harvested by centrifugation (4000 g, 10 min, 4 °C) and resuspended in 50 mM sodium phosphate, pH 8.0, 300 mM NaCl (buffer A) containing 1X EDTA-free protease inhibitors (Roche). Cells lysis was achieved adding lysozyme (final concentration of 1 mg/mL) followed by sonication. The cleared lysate obtained by centrifugation (18000 g for 45 min at 4 °C) was loaded onto a HisTrap® column (GE). After washing the column with 20 mM imidazole in buffer A, the recombinant fusion protein was eluted with a linear gradient of 20–500 mM imidazole in buffer A. Imidazole was removed from elution fractions containing the recombinant protein using a HiPrep 26/10 Desalting column (GE) equilibrated with buffer A and 5 mM dithiothreitol (DTT). Then, the fusion product was cleaved by adding a His-tagged 3C-type TEV protease^9^ at a 1:35–70 (mg/mL) protease:protein ratio (12 hours at 4 °C). Then, purification in a second HisTrap column renders tag-free FL 1CGrx1 in the flow through and resin-bound His-tagged *Ec*Trx and TEV. Untagged FL 1CGrx1 was concentrated and polished by size exclusion chromatography (SEC) on a HiLoad Superdex 75 (16/60) prep-grade column (GE) pre-equilibrated with 50 mM sodium phosphate pH 7.0, 150 mM NaCl, 3 mM DTT at 1 mL/min coupled to an AKTA FPLC (GE) with multi-wavelength detection. Additional N-terminal GAMG or GA residues derived from the cloning strategy are added to FL or truncated forms of 1CGrx1, respectively. About 40–50 mg of pure protein were obtained per liter of culture.

### Limited proteolysis assay

The truncated Δ64 1CGrx1 (residues L65-L184) was obtained by incubating FL 1CGrx1 with trypsin at a protease:protein ratio of 1:10.000 (w/w) in 20 mM Tris pH 7.8 100 mM NaCl for 30 min at 37 °C. The proteolytic conditions were optimized to obtain only the desired truncated protein, which has been unambiguously identified by mass spectrometry (for details, see Supplementary Data). Prior to the NMR analysis, the ^15^N-labeled Δ64 1CGrx1 was polished by size exclusion chromatography on a HiLoad Superdex 75 (16/60) prep-grade column (GE) pre-equilibrated with 50 mM sodium phosphate pH 7.0, 150 mM NaCl, 10 mM DTT.

### Multiangle light scattering (MALS)

FL and Δ76 1CGrx1 were analyzed by SEC-MALS as previously described^56^. The samples were loaded onto a Superdex 75 10/300 GL SEC column (GE-Healthcare) equilibrated with 20 mM Tris pH 8.0, 100 mM NaCl and 5 mM DTT connected to a multiple-angle laser light (690.0 nm) scattering DAWN EOS photometer (Wyatt Technology) and a refractive index detector (Optilab DSP, Wyatt). The specific refractive index increment (*d*n/*d*c) for the protein was taken as 0.185 mL/g^57^. The value of 1.331 for the solvent refractive index and the concentration of the eluted protein were determined using the refractive index detector. The weight average molecular masses, *M*_*w*_, were determined across the entire elution profile in the intervals of 0.2 s from MALS measurement using the ASTRA software (Wyatt Technology). A Rayleigh–Debye–Gans light scattering model was used to determine *M*_*w*_, using a Zimm plot. The uncertainties on *M*_*w*_ are a measure of the statistical consistency of the MALS data, obtained combining the standard deviations calculated for each slice in the analyzed peaks. Data analysis was performed using Astra version 5.3.4 following the manufacturer’s instructions.

### Nuclear magnetic resonance (NMR) of FL 1CGrx1

The preparation of FL 1CGrx1 for NMR analysis and the respective resonance assignment was previously reported^23^. Briefly, the samples for NMR experiments were prepared in 50 mM sodium phosphate, pH 7.0, 150 mM NaCl, and 10 mM DTT. Deuterated water (10% v/v), sodium 4,4-dimethyl-4-silapentane-1-sulfonate (DSS, 0.2 mM), EDTA (1 mM), sodium azide (0.05% w/v) and phenylmethylsulfonyl fluoride (PMSF, 0.2 mM) were finally added to the NMR tube.

### Residual dipolar coupling

^15^N-^1^H residual dipolar couplings (RDCs) were obtained from the difference of ^1^J_HN_ splittings measured in the presence and in the absence of filamentous phages Pf1 (ASLA biotech) at a concentration of 10 mg/mL. Measurement of ^1^J_HN_ splittings was carried out with the IPAP ^1^H, ^15^N-HSQC experiment^58^.

### Structure calculation

The resonance assignment of FL 1CGrx1 was previously reported^23^ and its structure was solved following the same procedure used to solve the structure of the truncated Δ76 version^8^. Briefly, backbone dihedral angle constraints were derived from ^15^N, ^13^C′, ^13^Cα,^13^Cβ, and Hα chemical shifts using TALOS+^35^. Distance restraints for structure determination were obtained from the ^15^N-edited and ^13^C-edited 3D NOESY-HSQC spectra. An automated method, based on the ATNOS/CANDID algorithms^59^, was used for NOESY peak-picking and assignment, implemented in the UNIO’10 v2.0.2^60^, combined with the torsion angle dynamics for structure calculation using CYANA 2.1^61^. The final distance restraint table and dihedral angle table were used to generate 100 conformers by a simulated annealing (SA) protocol using XPLOR-NIH 2.35 software. Briefly, SA protocol consisted of 5000 steps of dynamics at 3000 K and of 12000 steps of cooling from 3000 to 100 K. The SA procedure was followed by 250 cycles of Powell’s energy minimization. The 30 minimum-energy structures containing no distance and dihedral restraint violations were subjected to refinement by restrained molecular dynamics including RDCs in explicit water, with the AMBER package through the AMPS–NMR interface^62,63^. The default protocol of AMPS–NMR interface was used except for the initial step of energy minimization that was set to 10000. Only RDCs originated from well-defined secondary structure elements with heteronuclear NOE value greater than 0.6 were used. This filter resulted in 67 non-overlapped RDCs. Ten RDCs (around 15% of the total RDC) were randomly excluded in order to use them as a validation set after structural refinement. The remaining 57 RDCs were included in the refinement step with AMBER. The analysis of the structure and restraints were performed with *PDBStat* 5.10^64^. An RDC-based quality factor was calculated with the following formula proposed by Cornilescu and coworkers^37^ and using only the 10 RDCs excluded from the structure refinement:

$$Q=\sqrt{\frac{{\sum }_{i}{({D}_{i}^{exp}-{D}_{i}^{calc})}^{2}}{{\sum }_{i}{({D}_{i}^{exp})}^{2}}}$$

where ${D}_{i}^{exp}$ and ${D}_{i}^{calc}$ are, respectively, experimental and calculated dipolar couplings for the residues i. ${D}_{i}^{calc}$ values were back calculated with PALES^65^ using a fixed alignment tensor derived from the refined structure, by singular value decomposition best-fit of all selected RDC.

The assignment of the same construct is available on the Biological Magnetic Resonance Data Bank (BMRB; http://www.bmrb.wisc.edu/) with the Entry ID 19736^23^ and Protein Data Bank with entry 2MXN.

### Relaxation measurements

Backbone amide ^15^N longitudinal (T_1_) and transverse (T_2_) relaxation times and ^15^N{^1^H}-NOE were measured at 800 MHz (18.8 T) and 298 K. T_1_ and T_2_ were obtained by fitting cross peak volumes (I), measured as a function of the relaxation delay, to a single exponential decay using the Sparky software package^66^. NOE values were calculated as the ratio of peak volumes in spectra recorded with and without saturation. Delay times for R_1_ were: 10, 50, 100, 200, 400, 700, 1000, 1300, 1600 and 1000 ms. For R_2_ measurements, the following delays were employed: 16.31, 32.64, 48.96, 65.28, 97.92, 146.89, 179.52 and 228.5 ms.

### Pulse field gradient NMR

The pulsed field gradient (PFG) NMR self-diffusion measurements were performed at 600 MHz ^1^H frequency using a stimulated echo with bipolar gradients and a delay for eddy-current compensation^67^ at 298 K and with a protein concentration of 1 mM. Dioxane (20 μL 1% in D_2_O) was added to the sample as internal standard^34^. The length of all pulses and delays in the sequence were held constant and 20 spectra were acquired varying the strength of the diffusion gradient between 5% and 95% of its maximum value. The total pulse gradient width was 3.0 ms and the length of the diffusion delay was calibrated to give a total decay of 80–90% for signals. The translational diffusion coefficient of FL 1CGrx1 was determined by fitting the integrals of 10 different protein signals to an exponential function of the gradient strength and the reported value is an average of these values with the corresponding standard deviation^68^. The diffusion coefficient of the dioxane used as internal reference was determined by fitting the decay of its signal at 3.6 ppm and the same relative error as for the protein diffusion coefficient was assumed. The hydrodynamic radius of FL 1CGrx1 was calculated from the following equation and using dioxane as a reference (${R}_{h}^{Rif}$= 2.12 Å)^34^:

$${R}_{h}^{Prot}=\frac{{D}_{Rif}}{{D}_{Prot}}({R}_{h}^{Rif})$$

The compaction factor (C)was calculated, as proposed in ref.^34^, using the following formula:

$$C=\frac{{R}_{h}^{diso}-{R}_{h}}{{R}_{h}^{diso}-{R}_{h}^{fold}}$$

where ${R}_{h}^{diso}$ and ${R}_{h}^{fold}\,$are the predicted values of the hydrodynamic radii for the fully disordered and folded states, respectively^34^.

### Chemical shift perturbation

Amide chemical shift differences between FL- and Δ76–1CGrx1 was calculated using the following formula^69^:

$$\delta {{\rm{\Delta }}}_{AVE}=\sqrt{\frac{{{(}^{1}H{\rm{\Delta }}\delta )}^{2}+{(0.2{\times }^{15}N{\rm{\Delta }}\delta )}^{2}}{2}}$$

where ^1^H Δδ and ^15^N Δδ are the ^1^H and ^15^N amide chemical shift changes, respectively.

## Electronic supplementary material

Supplementary Information
