# Supplementary material for: The lineage-specific, intrinsically disordered N-terminal extension of monothiol glutaredoxin 1 from trypanosomes contains a regulatory region
Source: Sci Rep. 2018 Sep 12;8:13716. doi: 10.1038/s41598-018-31817-4 (PMC6135854; doi:10.1038/s41598-018-31817-4)
Supplement: Supplementary file 1 — Supplementary Information [file 41598_2018_31817_MOESM1_ESM.pdf]

The lineage-specific, intrinsically disordered N-terminal extension of monothiol glutaredoxin 1 from trypanosomes contains a regulatory region

Mattia Sturlese[‡‖], Bruno Manta[†£1], Andrea Bertarello[‡], Mariana Bonilla[†], Moreno Lelli[#○2], Barbara Zambelli[§], Karin Grunberg[†], Stefano Mammi[‡], Marcelo A. Comini[†] and Massimo Bellanda[‡*]

[‡]Department of Chemical Sciences, University of Padova, via Marzolo 1. 35131, Padova, Italy.

‖ Molecular Modeling Section (MMS), Department of Pharmaceutical and Pharmacological Sciences, University of Padova, via Marzolo 5, Padova, Italy.

[#] Department of Chemistry "Ugo Schiff", University of Florence, Via della Lastruccia 3, 50019 Sesto Fiorentino (FI), Italy.

[○]Magnetic Resonance Center (CERM), University of Florence, Via L. Sacconi 6, 50019 Sesto Fiorentino (FI), Italy.

[#] Centre de RMN à Très Hauts Champs, Institut des Sciences Analytiques (UMR 5280 - CNRS, ENS Lyon, UCB Lyon 1), Université de Lyon, 5 rue de la Doua, 69100 Villeurbanne, France.

§Department of Pharmacy and Biotechnology, University of Bologna, Viale Giuseppe Fanin 40, 40127 Bologna, Italy.

[†]Institut Pasteur de Montevideo, Mataojo 2020, 11400, Montevideo, Uruguay.

[£]Laboratorio de Fisicoquímica Biológica, Instituto de Química Biológica, Facultad de Ciencias, Universidad de la República. Igua 4425, 11400, Montevideo, Uruguay.

* Corresponding author: Dr. Massimo Bellanda, Department of Chemical Sciences, University of Padova, via Marzolo 1. 35131, Padova, Italy. massimo.bellanda@unipd.it.

[1]Current address: New England Biolabs, 240 County Road, Ipswich, MA 01938, USA

[2]Past address: Centre de RMN à Très Hauts Champs, Institut des Sciences Analytiques (UMR 5280 - CNRS, ENS Lyon, UCB Lyon 1), Université de Lyon, 5 rue de la Doua, 69100 Villeurbanne, France.

Supplementary Informations

Figure S1

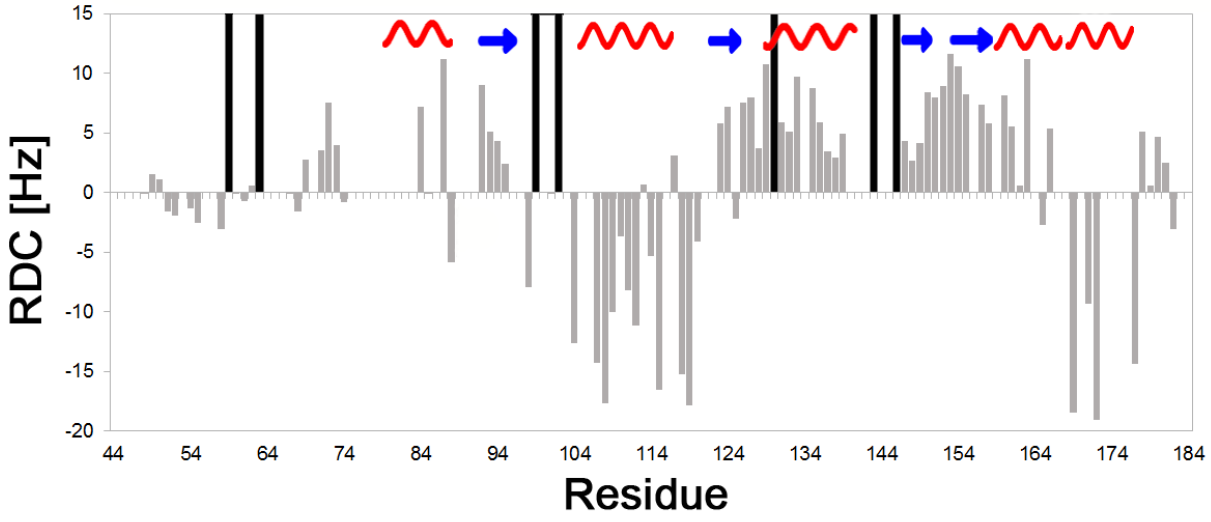

Plot of ^{1}H-^{15}N RDCs, measured in filamentous phages Pf1 (10 mg/mL) for 1CGrx1, as a function of the residue number. Residues with severe peak overlap or poor signal-to-noise ration were excluded; proline residues are indicated with black bars.

Figure S2

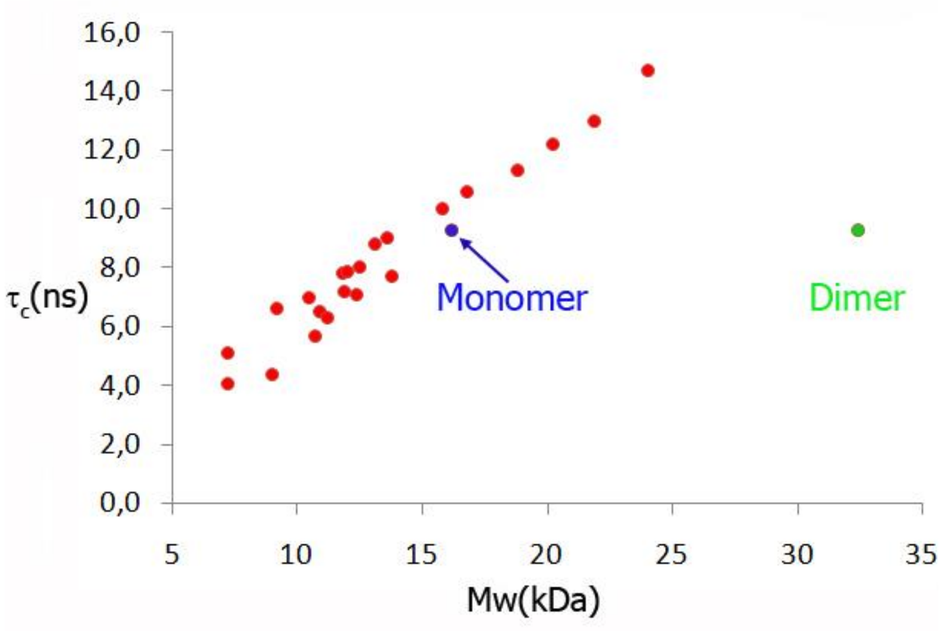

Plot of τ_c versus protein molecular weight (MW) obtained at the same temperature on a series of known monomeric proteins of varying size. Data for the plot (red dots) are from targets of the Northeast Structural Genomics Consortium (NESG) (www.nmr2.buffalo.edu/nesg.wiki/NMR_determined_Rotational_correlation_time).

The blue and green dots correspond to the correlation time measured in this work for Tb1CGrx1 and plotted at a molecular weight corresponding to a monomer (blue) or dimer (green).

Figure S3.

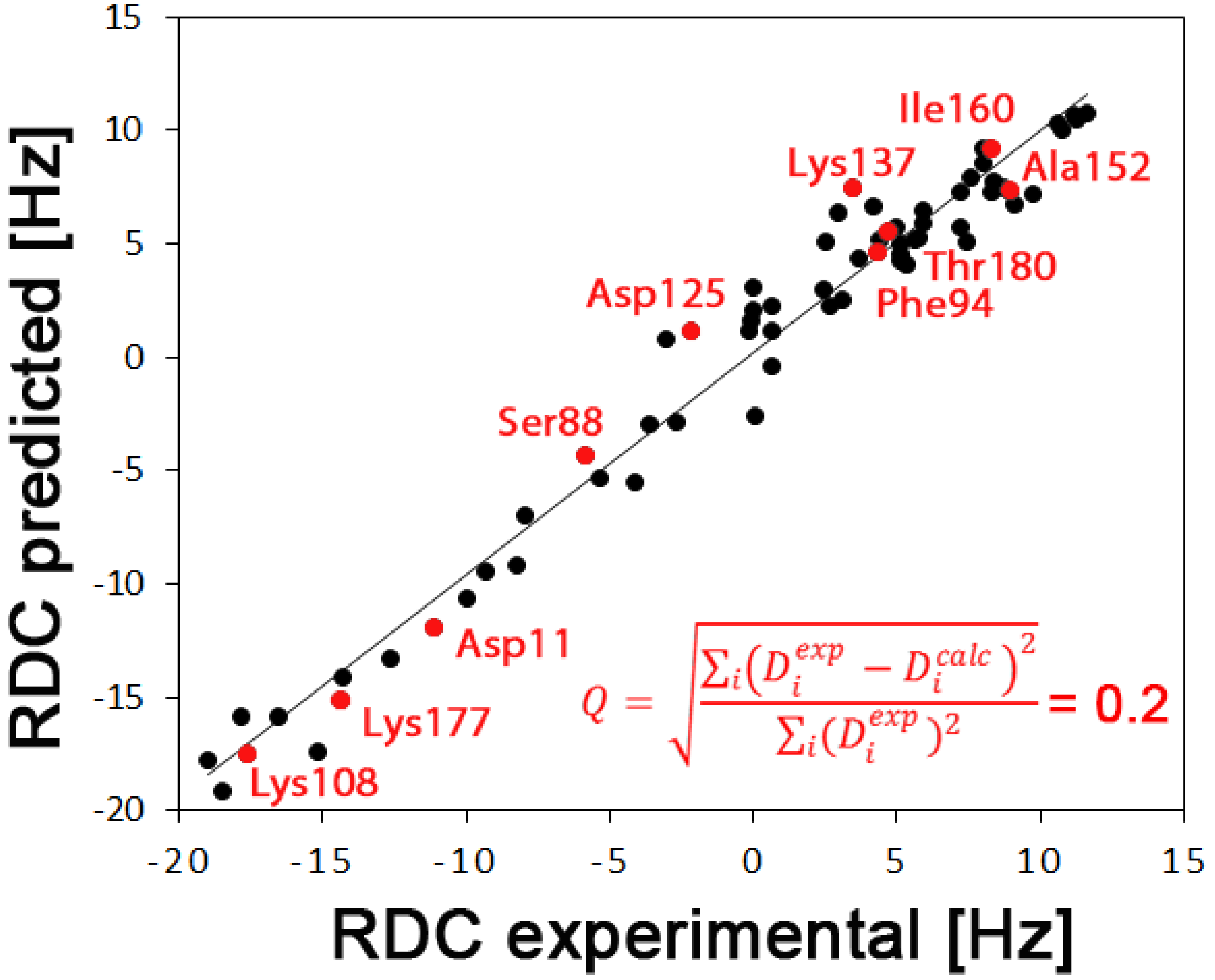

Comparison between experimental ^{1}H-^{15}N RDCs, measured in filamentous phages Pf1 (10 mg/mL), and values predicted from the NMR structure on 1CGrx1 presented in this paper (PDB code: 2MXN). The experimental RDCs corresponding to the residues indicated with red dots were not used for the refinement of the NMR structure and were employed to calculate the quality factor Q indicated in the insert.

Figure S4.

A

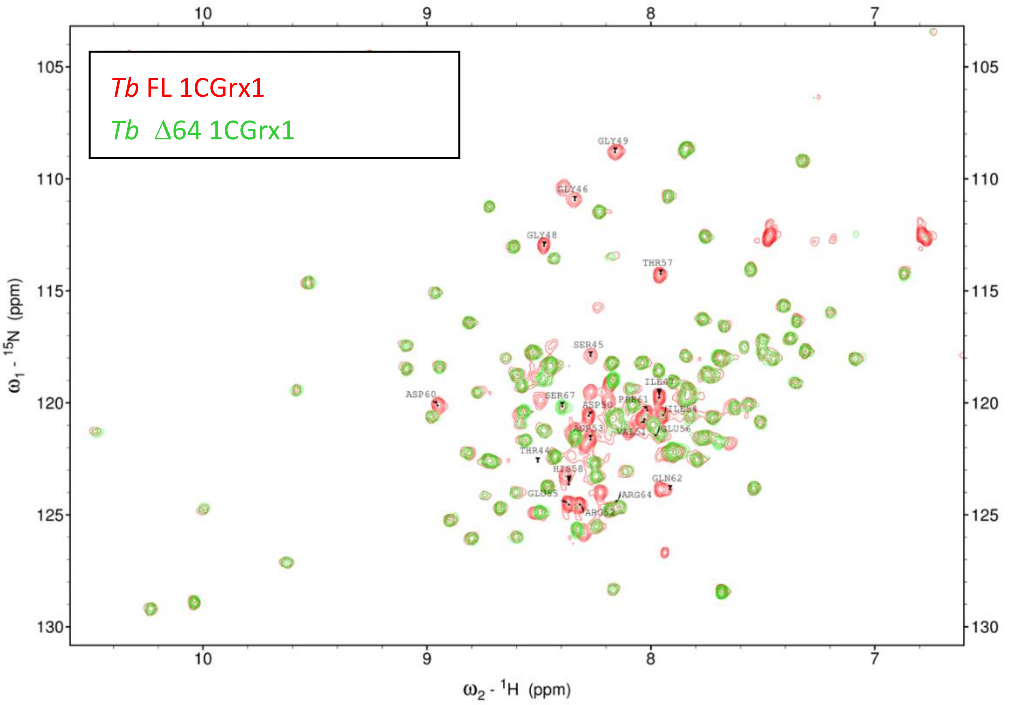

B

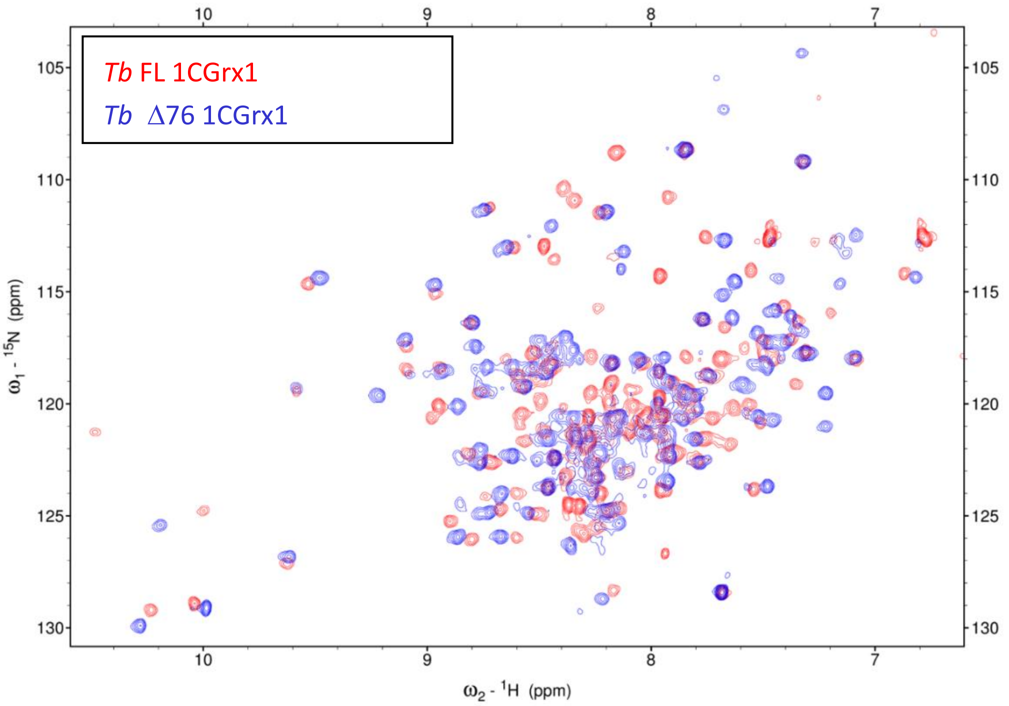

^{1}H-^{15}N-HSQC spectra of *Tb* FL 1CGrx1 compared to Δ64 truncated mutant obtained by limited proteolysis (panel A) and Δ76 truncated mutant (panel B).

Figure S5

A

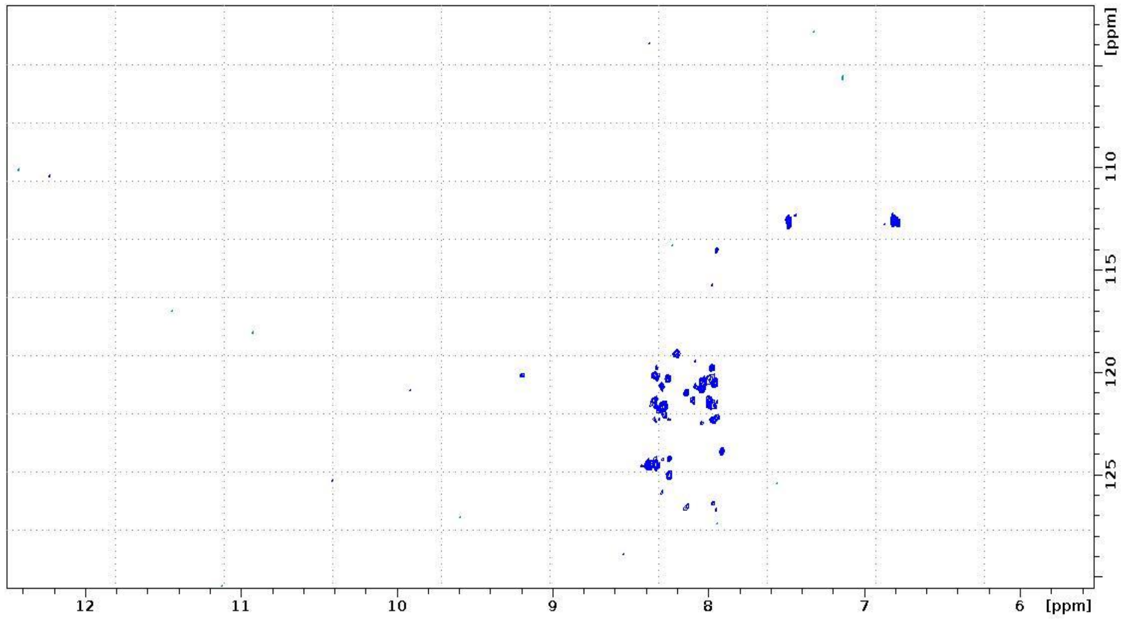

B

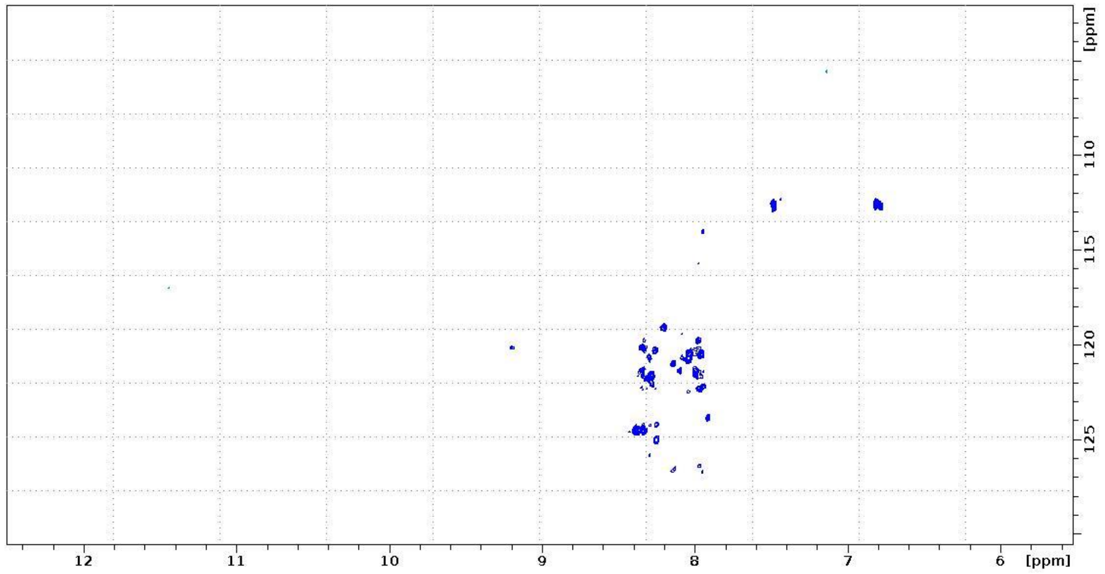

^{1}H,^{15}N-HSQC spectra of the “loop$^{(-)}$” (A) and of the “loop$^{(-)}$/HE” constructs derived from the *Tb* FL 1CGrx1 sequence (see text).

Trypsin digestion and spectrometry analysis of *Tb*1CGrx1

Proteolytic digestion of *Tb*1CGrx1 was achieved by incubating the protein with sequencing grade modified trypsin (Promega) at a protease:protein ratio of 1:10.000 (w/w) in 20 mM Tris pH7.8 100 mM NaCl for 45 min at 37°C. To stop the reaction 10 µL of 0.1% trifluoroacetic acid were added and this mix was cleaned using 0.1 % trifluoroacetic acid on C18 ZipTips (Millipore). Samples were eluted in a saturated solution of α-cyano-4-hydroxycinnamic acid in 0.1% trifluoroacetic acid in acetonitrile-H2O (60%, v/v) (matrix, see below).

At different time points, the reaction mixture was subjected to mass spectrometry analysis using a 4800 MALDI TOF/TOF instrument (Applied Biosystems) in positive ion reflector or linear mode with a matrix solution of α-cyano-4-hydroxycinnamic acid in 0.1% trifluoroacetic acid in acetonitrile-H2O (60%, v/v). External calibration was performed using a mixture of standard peptides (Applied Biosystems). Untreated *Tb*1CGrx1 was run as a control. Data were compared with the theoretical masses of the peptides. For sequence analysis, MS/MS spectra of selected precursor (N-terminal peptide released from trypsin cleavage) was acquired and compared with theoretical fragmentation.

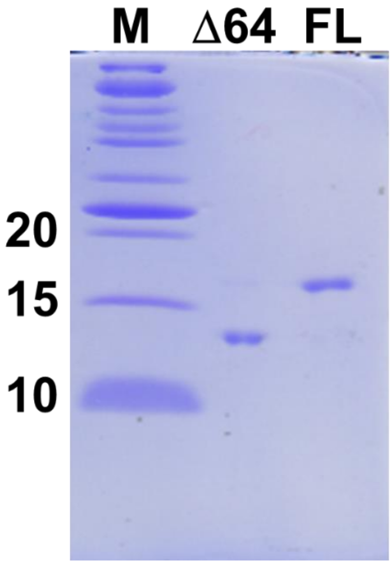

SDS-PAGE (17 % gel) of trypsin-treated FL *Tb*1CGrx1. Samples from trypsin-treated (30 min at 37°C; Δ64) and non-treated (FL) *Tb*1CGrx1 were separated on a SDS-PAGE under reducing conditions. M, molecular weight marker (P7704, New England Biolabs Inc.).

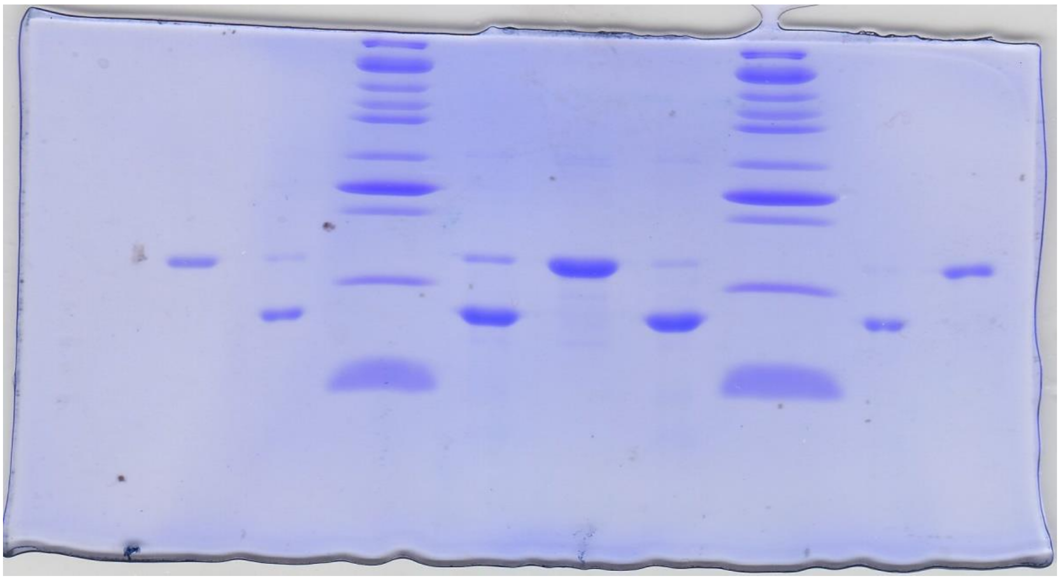

Full length SDS-PAGE (17 % gel) of trypsin-treated FL *Tb*1CGrx1. The last three lines were used for the previous figure. Lane 1: 0.89 ug 1CGrx1, time 0 min;lane 2: 0.89 ug 1CGrx1, time 30 min (trypsin); lane 3: protein ladder; lane 4: 4.42 ug 1CGrx1, time 30 min (trypsin); lane 5: 4.42 ug 1CGrx1, time 0; lane 6: 4.42 ug 1CGrx1, time 45 min (trypsin); lane 7: protein ladder; lane 8: 0.89 ug 1Cgrx1, time 45 min (trypsin); lane 9: 0.89 ug 1CGrx1, time 0.

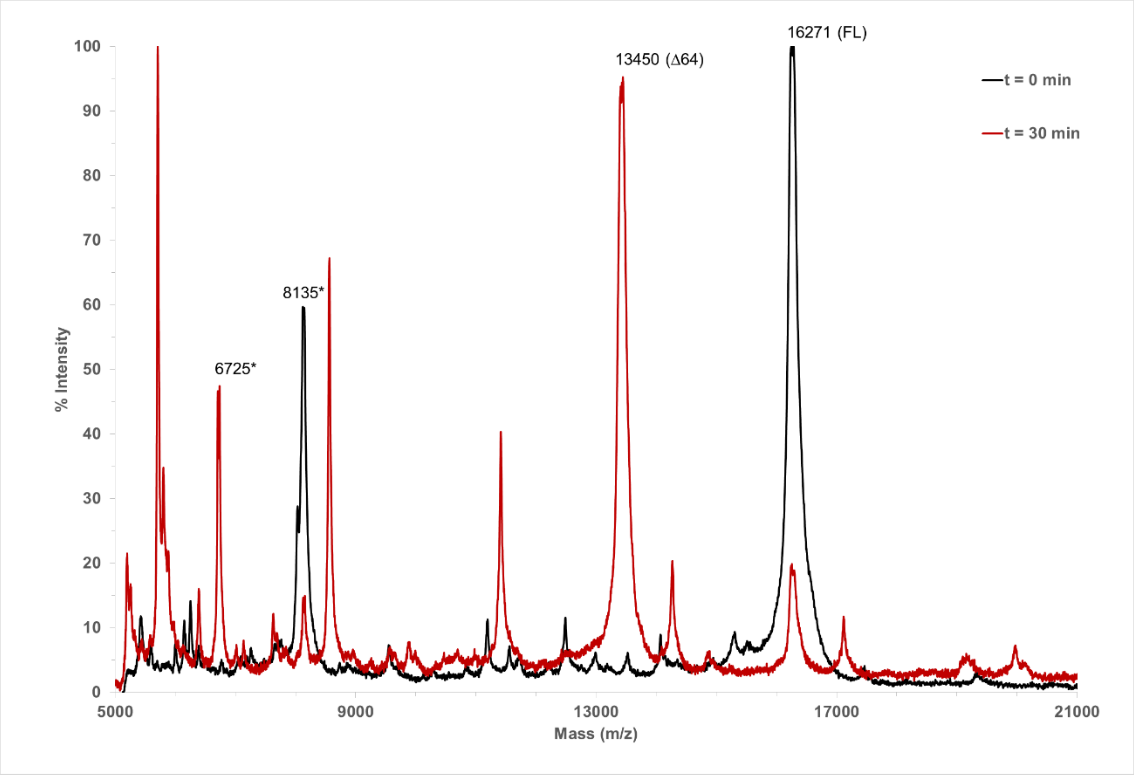

Mass spectrometry analysis (linear mode) of tryptic digest of FL *Tb*1CGrx1. The mass spectra of non-treated (black line) and trypsin-treated (30 min; red line) full-length *Tb*1CGrx1 are shown. The mass peak corresponding to full-length (FL) and trypsin-cleavage polypeptide at Arg64 (Δ64) is shown. With asterisk is indicated the mass peak of the corresponding di-charged masses of FL and Δ64 *Tb*1CGrx1.

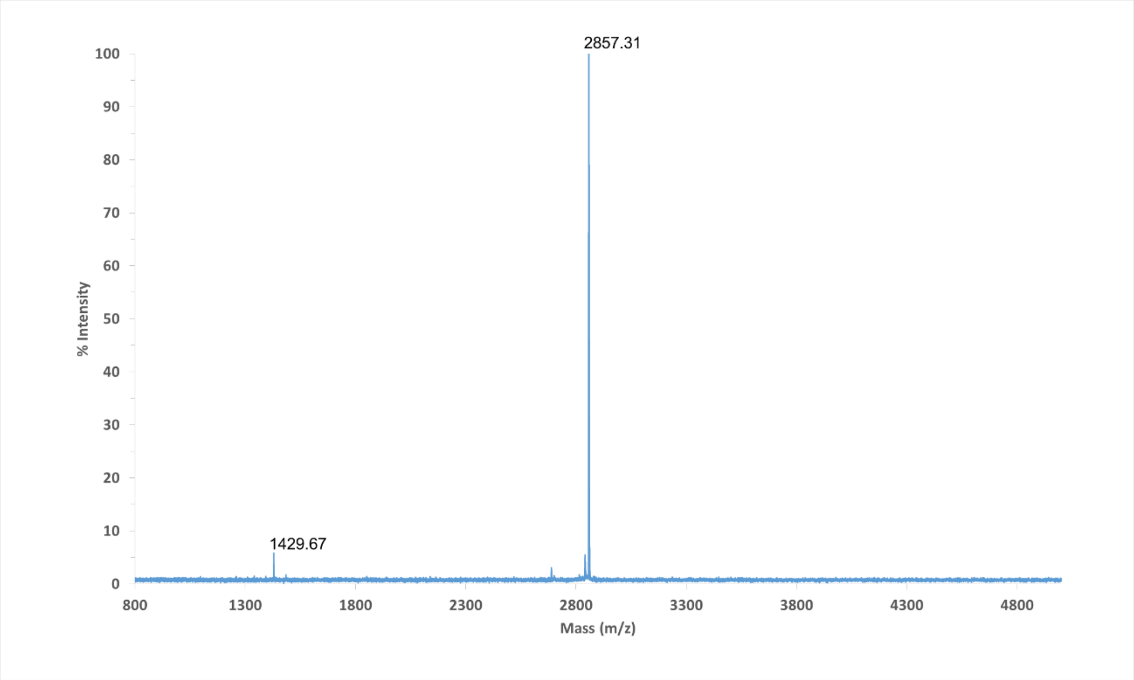

Mass spectrometry analysis (reflector mode) of N-terminal peptide released from trypsin treated FL *Tb*1CGrx1. The mass peaks corresponding to the N-terminal peptide of full-length GAMQ^{42}-R^{64} and its di-charged form are shown.

GPMAW v. 4.02 (32-bit) - [MS/MS fragmentation - C118 H185 N37 O44 S1]

File Edit Setup Search Utilities Window Help

SS Aa_mass.mss

GAMGQSTSGIGGDVRDIEETHPDFQPR 2856.3097 Da Mo S MS N: Hydrogen C: Free acid

a	b	c''		x	y''	z
30.034	58.029	109.061	1 Gly 27	-	-	-
101.071	129.066	180.099	2 Ala 26	2826.275	2800.296	2781.254
232.112	260.107	311.139	3 Met 25	2755.238	2729.259	2710.217
289.133	317.128	368.160	4 Gly 24	2624.198	2598.218	2579.176
417.192	445.187	496.219	5 Gln 23	2567.176	2541.197	2522.155
504.224	532.219	583.251	6 Ser 22	2439.118	2413.138	2394.096
605.272	633.267	684.299	7 Thr 21	2352.086	2326.106	2307.064
692.304	720.299	771.331	8 Ser 20	2251.038	2225.059	2206.016
749.325	777.320	828.352	9 Gly 19	2164.006	2138.027	2118.984
862.409	890.404	941.436	10 Ile 18	2106.984	2081.005	2061.963
919.431	947.426	998.458	11 Gly 17	1993.900	1967.921	1948.879
976.452	1004.447	1055.479	12 Gly 16	1936.879	1910.900	1891.857
1091.479	1119.474	1170.506	13 Asp 15	1879.857	1853.878	1834.836
1190.548	1218.542	1269.575	14 Val 14	1764.830	1738.851	1719.809
1346.649	1374.644	1425.676	15 Arg 13	1665.762	1639.783	1620.741
1461.676	1489.671	1540.703	16 Asp 12	1509.661	1483.682	1464.639
1574.760	1602.755	1653.787	17 Ile 11	1394.634	1368.655	1349.612
1703.802	1731.797	1782.829	18 Glu 10	1281.550	1255.571	1236.528
1832.845	1860.840	1911.872	19 Glu 9	1152.507	1126.528	1107.486
1933.893	1961.887	2012.920	20 Thr 8	1023.465	997.486	978.443
2070.951	2098.946	2149.979	21 His 7	922.417	896.438	877.396
2168.004	2195.999	2247.031	22 Pro 6	785.358	759.379	740.337
2283.031	2311.026	2362.058	23 Asp 5	688.305	662.326	643.284
2430.100	2458.094	2509.127	24 Phe 4	573.278	547.299	528.257
2558.158	2586.153	2637.185	25 Gln 3	426.210	400.231	381.189
2655.211	2683.206	2734.238	26 Pro 2	298.151	272.172	253.130
2811.312	2839.307	2890.339	27 Arg 1	201.099	175.120	156.077

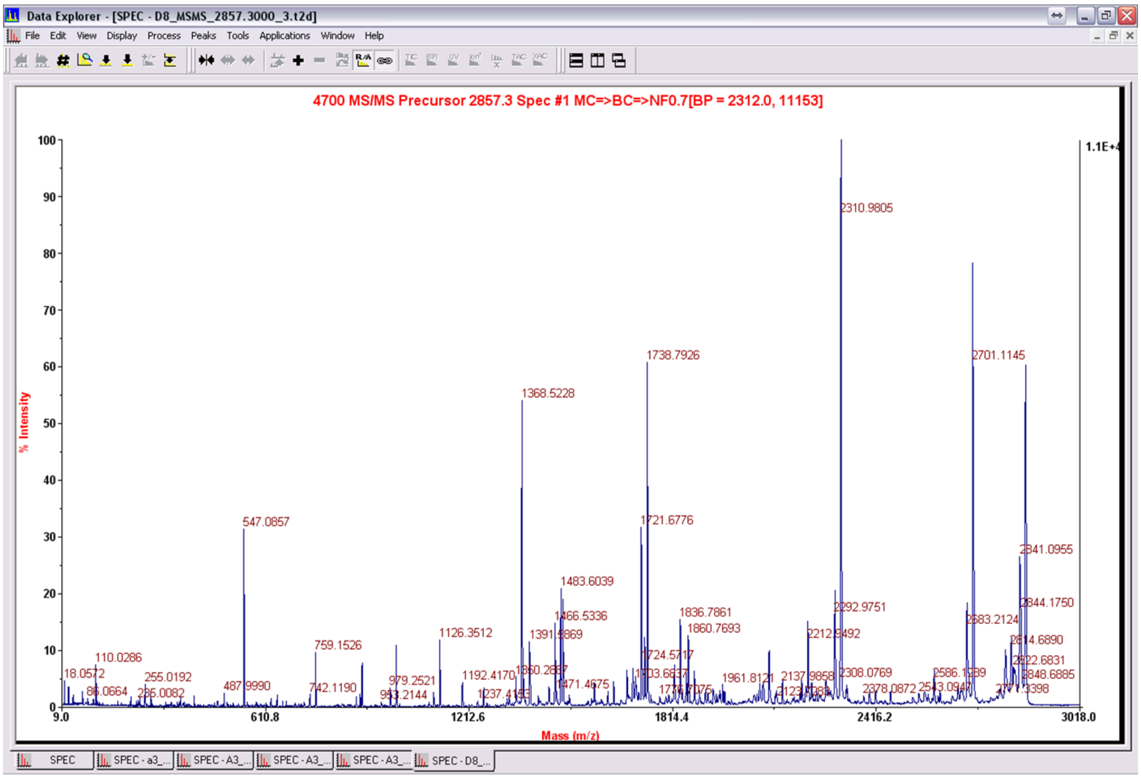

MS/MS fragmentation analysis of N-terminal peptide released from trypsin treated FL *Tb*1CGrx1. The theoretical (table) and observed (mass spectra) masses corresponding to the fragmentation of the N-terminal peptide (GAMQ^{42}-R^{64}, 2857.3 Da) are shown.
